# Differences in Olfactory Performance and Neuropsychiatric Symptoms in Subjective Cognitive Decline

**DOI:** 10.1002/alz70857_098030

**Published:** 2025-12-24

**Authors:** Olivier Fortier‐Lebel, Émilie Hudon, Sarah Brosse, Benjamin Boller, Johannes Frasnelli

**Affiliations:** ^1^ Université du Québec à Trois‐Rivières, Trois‐Rivières, QC, Canada; ^2^ Research Center of the Hôpital du Sacré‐Cœur de Montréal, Montréal, QC, Canada; ^3^ Institut Universitaire de Gériatrie de Montréal, Montréal, QC, Canada; ^4^ Université de Montréal, Montréal, QC, Canada

## Abstract

**Background:**

The presence of olfactory dysfunction and neuropsychiatric symptoms, such as anxiety and depression, is believed to be strongly associated with the progression from mild cognitive impairment (MCI) to Alzheimer's Disease (AD). However, less is known if such impairments are already present in subjective cognitive decline (SCD) — a preclinical stage of AD characterized by self‐reported memory complaints and no objective cognitive deficits on standard neuropsychological tests. This study aims to characterize olfactory function, anxiety, and depression in individuals with SCD, compared to healthy controls.

**Method:**

A total of 110 participants aged 60 and older were recruited, including 59 with SCD (42 women) and 51 healthy controls (35 women). Participants completed anxiety (GAI) and depression (GDS) questionnaires and underwent olfactory testing with the *Sniffin’ Sticks* battery (threshold, discrimination, and identification).

**Result:**

The SCD group exhibited significantly lower global olfactory performance than controls. Anxiety and depression scores were significantly higher in the SCD group compared to healthy controls. No significant differences were found in specific olfactory submeasures. Combining global olfactory performance with anxiety and depression scores improved SCD status prediction and classification accuracy.

**Conclusion:**

These findings highlight subtle but significant and objective lower olfactory performance in individuals with SCD. Olfactory event related potentials should be explored to better understand possible underlying neurophysiological mechanisms.

